# Two-stage mural image restoration using an edge-constrained attention mechanism

**DOI:** 10.1371/journal.pone.0307811

**Published:** 2024-09-06

**Authors:** Jianfang Cao, Xianhui Wang, Fang Wang, Zhen Cao, Jiaqi Liu, Zhuolin Yang

**Affiliations:** 1 Department of Computer Science and Technology, Xinzhou Normal University, Xinzhou, China; 2 School of Computer Science and Technology, Taiyuan University of Science and Technology, Taiyuan, China; Yunnan University, CHINA

## Abstract

The current mainstream image restoration methods have difficulty fully learning the structure and color information of murals in mural image restoration tasks due to the limited size of the available datasets, resulting in problems such as structural loss and texture errors. This study proposes a two-stage mural restoration network based on an edge-constrained attention mechanism. This paper introduces additional sketches as inputs during the coarse restoration phase and incorporates a local edge loss function to enable the network to generate corresponding structural information based on the sketches. In the fine restoration phase, the calculation for the similarity between missing areas and known areas is optimized to enhance the consistency of the restoration results with the texture of the known areas. Furthermore, a structure-guided attention propagation block is introduced after adopting the attention mechanism. This block selectively integrates surrounding contextual information to update the attention score map, thereby enhancing the coherence and plausibility of the generated textures. The experimental results show that the proposed method outperforms the current mainstream restoration methods according to various assessment indices. The proposed method generates high-quality structural information according to user guidance information, and the repaired texture is highly visually consistent with that of the original mural, with few noticeable deviations. This study provides a new approach for mural restoration, which may positively impact cultural heritage protection and artistic restoration applications.

## Introduction

Ancient murals contain a large amount of historical and cultural information that thoroughly reflects the artistic style of each period and how this style evolved; moreover, such murals reflect the historical integration of Chinese and Western art [1–3]. However, due to weathering and human destruction, many murals have fractured, faded or been damaged in other ways. Traditional mural restoration methods usually involve directly painting these cultural relics. However, the workload of these methods is high, the restoration time is long, and the restoration method is irreversible, which may cause further mural damage. In contrast, digital restoration methods are typically low risk, highly efficient and enable permanent preservation. Therefore, the use of digital image restoration technology for virtual restoration is a topic of interest among both domestic and foreign scholars.

Traditional methods for image restoration can be primarily either based on texture block matching or sparse representations. These methods are usually based on diffusion or image block matching mechanisms [4–7]. With this type of method, the low-level features of an undamaged area are transferred to the damaged area, and favorable results have been obtained for images with small damaged areas, simple structures or repeated textures. However, with this approach, the damaged areas are repaired based on the local features of a single image; thus, the true content cannot be expressed, and the semantics of the repair results are unreasonable.

As deep learning develops, its notable generalizability has provided a new idea for image repair, which can be adopted to repair the overall image content at the semantic level. The context encoder [8] is one of the earliest deep learning methods used for semantic image restoration. In this method, an autoencoder structure is used to repair the defect area by minimizing the pixel-level reconstruction loss and adversarial loss. However, the context encoder method has several limitations, including the information bottleneck introduced by the fully connected layer and the lack of constraints on the local image area, resulting in obvious visual artifacts in the inpainting area of the output image. To address the information bottleneck problem of the context encoder method, Iizuka et al. [9] reduced the number of downlink sampling layers and applied a series of dilated convolutional layers to replace the fully connected layer. In addition, they introduced a local discriminator to improve the quality of the restored image. Yang et al. [10] introduced coarse-to-fine convolutional network schemes for image inpainting. Yan et al. [11] added a shift connection layer based on U-Net [12] and then guided the encoder to repair the features of the unknown region via feature fusion. Yu et al. [13] introduced the attention mechanism in the fine repair stage. In their method, features that are similar to those of the region to be repaired are extracted from the known region by the context attention layer, and the damaged region is then constructed according to the similarity scores of the blocks to ensure that the network is guided by full-text information. Liu et al. [14] proposed the coherent semantic attention (CSA) mechanism, which took into consideration the association between the known and damaged regions but that between the internal units within the missing region. Their method effectively addresses the issues of internal faults and distortions in the repair area, which improves image repair effect to a new high level. Although these methods can generate relatively fine textural details, structural distortions may occur. Nazeri et al. [15] proposed a two-stage restoration method that first predicts and generates contour lines to obtain complete boundary information and then employs this information to guide image color filling. This method addresses the structural distortion problem to some extent; however, this approach has difficulty directly predicting the complete contours of the missing regions for murals with complex structures. Furthermore, incorrect structural information generated by the model cannot be modified by the user as needed. To introduce user prior knowledge to guide repair, Portenier et al. [16] proposed the Faceshop model, which combines image generation with image translation; in this model, external sketches are employed to achieve end-to-end training. Yu et al. [17] replaced the traditional convolution with the gated convolution to distinguish masks and user-provided sketch information. The network generates the corresponding image content according to the sketch information. Ren et al. [18] introduced gated convolutions into Thang-ga image restoration and integrated image edge information extracted by the Canny algorithm as an extra input during training. Their method improved the restoration of structural information in mural images. To further enhance restoration quality, Liu et al. [19] proposed the mural image restoration model MPR-Net, which uses multistage progressive inference from global to local receptive fields to address the issue of semantic singularity in recurrent networks, achieving detailed texture restoration.

In the past three years, attention-based image restoration models [20,21] have been further developed, which generate images with more refined textures. Li et al. [22] proposed a cyclic feature inference network, which embeds the attention mechanism into the recurrent feature reasoning (RFR) module; the resulting model can be generalized by repeatedly using the parameters of the RFR module. In addition, they introduced the knowledge-consistent attention module to fuse the attention score in an adaptive manner, which gradually improves the feature map, leading to better texture restoration effects in a wide range of missing areas. Cao et al. [23] developed a multilevel attention propagation-driven image restoration network. This network compresses high-level features extracted from full-resolution images into multiscale compact features and then uses these compact features for multilevel attention feature propagation based on the scale of the features, enabling the propagation of advanced features (including structural and detail features) in the network. Chen et al. [24] proposed an image restoration model based on the combination of structure-constrained attention (SCA) and a pixel-level CAM module, which introduces CSA in the coarse repair stage to enhance semantic-level restoration and pixel-level CA in the fine repair stage to enhance pixel-level restoration. All these methods leverage the coarse repair results of the damaged area and the true values of the known area; however, these approaches cannot precisely determine the pixel relationship between the damaged and known areas. Thus, when these approaches are applied for mural image restoration, noticeable texture restoration deviations can occur.

Therefore, a two-stage mural restoration network based on edge-constrained attention propagation is proposed in this study. The main contributions can be summarized as follows:

1. The existing methods for feature extraction may produce model outputs that do not satisfy the requirements for sketch information. In view of this issue, a local structure loss function, L_edge_, is proposed. In addition, the cross-entropy loss is used to constrain the distance between the pixel distribution of the generated structural information and that of the prior structural information, thus guiding the generator to generate reasonable and clear structures based on the structural information provided by the user.
2. The existing methods employing attention mechanism calculate similarity between the generated results of the to-be-repaired region and the original information from the known region. This approach demands stringent criteria for achieving satisfactory outcomes in first-stage repairs, often leading to texture errors in the final repair results. To mitigate this issue, an SCA module is designed, which enables the attention score to more accurately reflect the degree of correlation between the known and unknown regions, thereby improving the texture consistency between the generated and known regions.
3. For mural images with complex structures, first-stage repair may not accurately infer the correct repair result based on semantic information, potentially resulting in errors in the final repair result. To address this challenge, a structural-constrained attention propagation (SCAP) layer is designed, which enables the network to infer and update attention scores based on sketch structural information, thereby generating more reasonable semantic information.

## Methodology

### Overall network design

**Fig 1:**
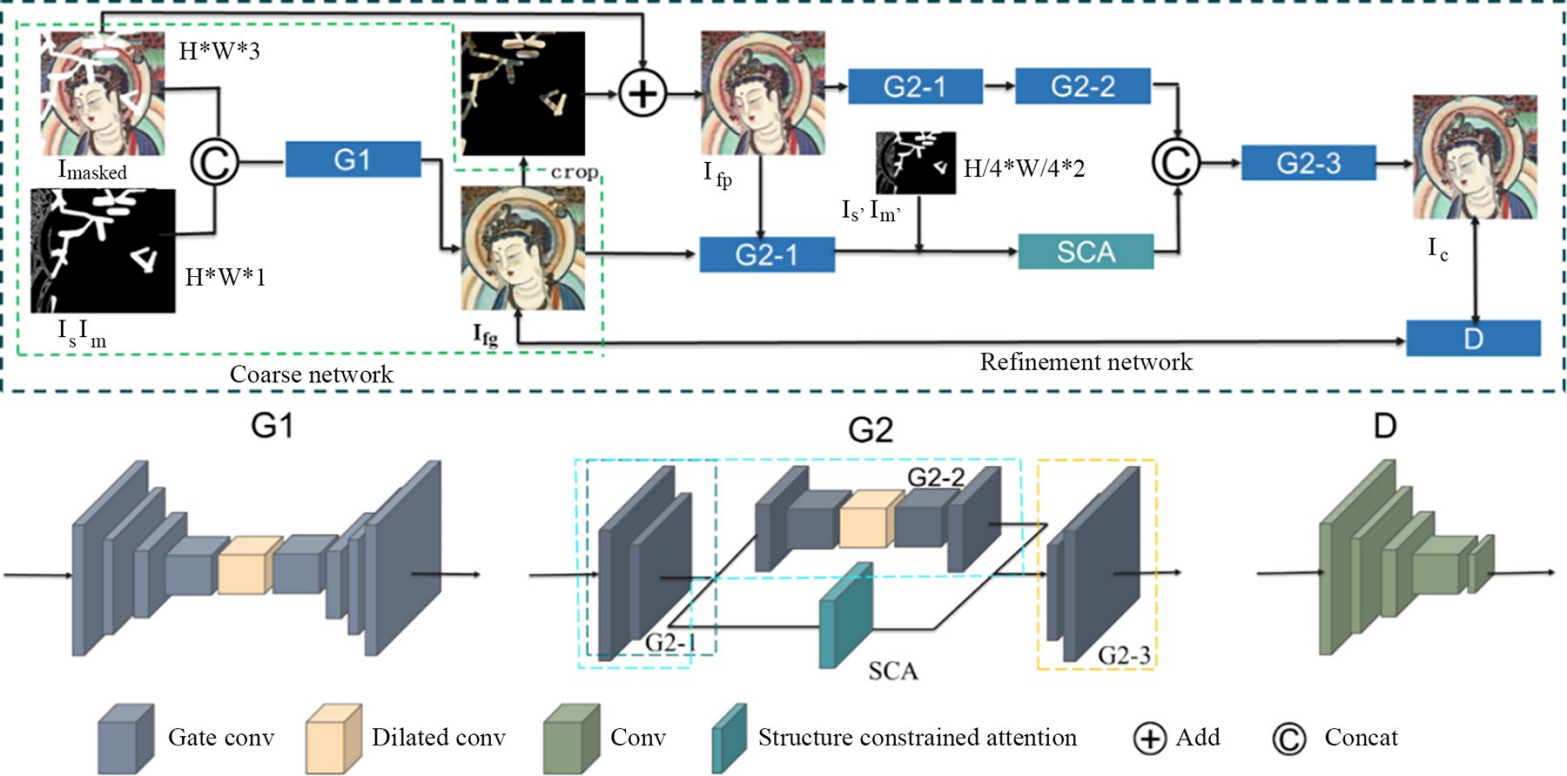
Overall design of the structure-constrained attention network (SCAnet).

The network repair process and overall structure of the two-stage mural image restoration method based on the proposed SCA mechanism are shown in Fig 1. The restoration process of the model consists of two main stages: coarse and fine repair. In the coarse repair stage, channel mosaicking is performed for I_masked_ (the image to be repaired), I_s_ (the sketch containing the structural information of the missing areas) and I_m_ (the mask of the area to be repaired); then, image I_fg_ is obtained through generator G1, and the areas in this image are all generated. Next, I_fg_ is cropped and added to the damaged area of I_masked_, yielding I_fp_. In the fine repair stage, I_fp_ is processed by generator G2-1, with one part used as the input for G2-2 and the other part combined with I_s’_ (obtained through 4× downsampling of the bilinear interpolation of I_s_) and I_m’_ (obtained through 4× downsampling of the bilinear interpolation of I_m_), which is used as the input to the lower branch of the SCA module. The outputs of the two branches are spliced, and the final restoration result I_c_ is obtained through generator G2-3. The main components of the network include generator G1, generator G2 and discriminator D. The structure of G1 is summarized in Table 1. The upper branch of G2 has the same structure as G1, the lower branch of G2 includes the edge-constrained SCA module (this module is detailed in a later section), and the discriminator D is composed of five 5×5 ordinary convolutions with a step size of 2, with output channel numbers of 48, 96, 192, 192 and 1.

**Table 1:** The structure of G1.

Type	Patch size/stride	Output size
Gate conv	5×5/2	H/2×W/2×48
Gate conv	3×3/2	H/4×W/4×96
Gate conv	3×3/2	H/8×W/8×192
Gate conv	3×3/1	H/8×W/8×192
Dilated conv	3×3/1	H/8×W/8×192
Gate conv	3×3/1	H/8×W/8×192
Transpose conv	3×3/1	H/4×W/4×96
Transpose conv	3×3/1	H/2×W/2×48
Transpose conv	3×3/1	H×W×3

#### Coarse repair network

The role of the coarse repair network is to fill the missing area with the appropriate structure and texture and determine the similarity between the missing and known areas, which is used by the fine repair network. The network includes multiple downsampling modules, and dilated convolutions are added to expand the receptive field of the network, thereby allowing the network to focus on the known global information. To address the issue that ordinary and partial convolutions cannot consider the pixels in the missing area as invalid pixels and cannot process the sketch information as effective pixels, gated convolutions are introduced to replace the ordinary convolutions. By using gated convolutions, the influence of missing regions is reduced in the feature extraction process, and the guidance information of the sketches is highlighted. The gated convolution can be expressed as follows:

$$Feature_{y,x}=\sum \sum W_{f}\cdot I$$ (1)

$$Gating_{y,x}=\sum \sum W_{g}.I$$ (2)

$$O_{y,x}=\phi (Feature_{y,x})⊙\sigma (Gating_{y,x})$$ (3)

where I denotes the input, which is the feature after downsampling in the network; W_g_ and W_f_ are the convolution filters used to calculate the gating values and eigenvalues, respectively; *ϕ* is the rectified linear unit (ReLU) activation function; and σ is the sigmoid activation function, which outputs the gating value within the range of [0,1]. Through the gating value, the user guidance information and missing area information can be separated to highlight the guidance information of the sketch and ensure the accuracy of the structural information in the repaired region.

#### Fine repair network

The fine repair network is used to further optimize the coarse repair results, eliminate the texture smoothing problem from the coarse repair stage, and ensure consistency between the repaired and known areas. The fine repair network uses two parallel repair branches that consider structural and textural information. Deep learning methods have difficulty achieving fine texture restoration due to issues such as small mural datasets, complex colors in murals, and differences between the distributions of the strong internal features of the images. In this paper, the SCA mechanism is proposed, which obtains the correlation between the missing and known regions by generating a coarse result. An attention propagation block based on structural constraints is added to update the correlation, and finally, fine texture details are reconstructed.

#### Discriminator

The spectral-normalized PatchGAN (SN-PatchGAN) algorithm is used in the discriminator network. The discriminator network comprises five standard convolutions with a convolution kernel size of 5×5 and a step size of 2. Since the SN-PatchGAN discriminator network aims to perform feature extraction and true–false discrimination for each image block, the local textural details of the mural can be learned and enhanced by this network.

#### Loss function

The loss functions used in this paper include the adversarial loss, pixel reconstruction loss, perceptual loss and local edge loss.

The adversarial loss can improve the visual quality of the generated image and is often used in image generation [25] and image style transfer [26] tasks. In addition, the adversarial loss aims to continuously optimize the generator and discriminator to improve the detailed information of the generated image. The adversarial loss can be expressed as follows:

$$L_{\mathrm{adv}}=\underset{\Theta }{min}\;\underset{D}{max}\;E_{I^{gt}∼p_{\mathrm{data}}(I^{gt})}[lg\;D(I_{gt})]+E_{I^{\mathrm{out}}∼p_{\mathrm{miss}}(I_{\mathrm{pred}})}[\mathrm{lg}\left(1-D(I_{\mathrm{pred}})\right)]$$ (4)

where P_data_(I_gt_) is the distribution of the real image, and P_miss_(I_gt_) is the distribution of the repaired image. The generator aims to minimize the results as much as possible, while the discriminator aims to maximize the results as much as possible. In this way, the model can be continuously optimized.

The pixel reconstruction loss is used to measure the pixel differences between the generated mural *I*_pred_ and the real mural *I*_*gt*_, which can be expressed as follows:

$$L_{l1}={∥I_{\mathrm{pred}}-I_{gt}∥}_{1}$$ (5)

In the perceptual loss function, the features obtained by the convolutions are compared with those of the real image. This loss function can be used to measure the high-level semantic similarity between images [27] and effectively improve the structure of the repaired image. The perceptual loss can be expressed as follows:

$$L_{\mathrm{perc}}=\sum_{l=1}^{N}\frac{1}{h_{l}w_{l}c_{l}}{∥\phi_{i}(I_{gt})-\phi_{i}(I_{\mathrm{pred}})∥}_{2}$$ (6)

where *ϕ*_*i*_ is the *l*-layer feature map of image I extracted from the pooling layer of the VGG-16 [28] network pretrained based on the ImageNet [29] dataset, and *h*_*l*_, *w*_*l*_ and *c*_*l*_ are the length, width and number of channels for *ϕ*_*i*_(*I*), respectively.

The local edge loss (L_edge_) is a new loss function proposed in this paper to measure the pixel difference between the structural information of the generated region and the sketch. This loss function is explained in detail in the Innovation section.

To focus each generator on the tasks that need more attention in each stage, the two generators are separately trained. In the coarse repair stage, reasonable structural information is generated based on the sketch information, and the correlation between the defect and known areas is determined. The loss function for this stage can be expressed as follows:

$$L_{coar}=\alpha L_{l1}+(1-\alpha )L_{edge}$$ (7)

where *α* is a parameter used to focus on certain areas in the image, which addresses issues such as incompleteness and errors in the restored images.

The fine repair phase aims to generate finer and more realistic texture information than that generated in the coarse repair stage. The loss function for this stage can be expressed as follows:

$$L_{refi}={\lambda_{\mathrm{adv}}L_{adv}+\lambda }_{l1}L_{l1}+\lambda_{\mathrm{perc}}L_{\mathrm{perc}}$$ (8)

### Improvements

#### The local edge loss function L_edge_

As the network depth increases, the network focuses more on the details of the image and less on the structural information contained in the sketch, which reduces the guiding role of the sketch. Moreover, the generated structural information is blurred when only gated convolutions are used. Therefore, a local edge loss L_edge_ is proposed in this paper to further constrain the network repair process. In the proposed loss function, I_gt_ denotes the intact mural image, and I_s_ denotes the edge detection map of I_gt_, where 1 represents an edge position and 0 represents a nonedge position. Moreover, I_m_ is the normalized mask map, with 0 denoting missing areas and 1 denoting known areas. I_s_ and I_m_ are dot-multiplied to obtain sketch E, which denotes the user-provided guidance structural information for the missing area. Then, the Canny edge detection algorithm is used to extract structural information I_f_ from the coarse-stage generation result to obtain the structural information graph Y. The proportion of correct pixel points in Y considering the user-guided information is calculated as the edge loss:

$$loss_{edge}=\frac{\sum_{x=1}^{W}\sum_{y=1}^{H}E_{i,j}\cdot (\left|Y_{i,j}-E_{i,j}\right|)}{\sum_{x=1}^{W}\sum_{y=1}^{H}E_{i,j}}$$ (9)

where Y_i,j_ is the predicted structural information for pixel (i, j), and E_i,j_ is the true value of the structural information for pixel (i, j) in the missing area. The numerator represents the number of edge pixels with matched structural information between the generated result and user-guided structure, and the denominator represents the number of edge pixels used to guide the structural information. The loss function ranges from 0 to 1. For each pixel in the user guidance information area, the predicted value should be as close as possible to the true value of the edge image. If a pixel is present in the nonuser guidance information area, we do not consider the corresponding loss to prevent imposing unnecessary restrictions on the nonuser guidance area in the repair process.

The edge loss function and the original repair loss function are weighted and summed to obtain the total loss function. Then, gradient descent and other optimization methods are used to minimize the total loss function, and finally, the optimal repair result is obtained.

#### Proposed SCA mechanism

**Fig 2:**
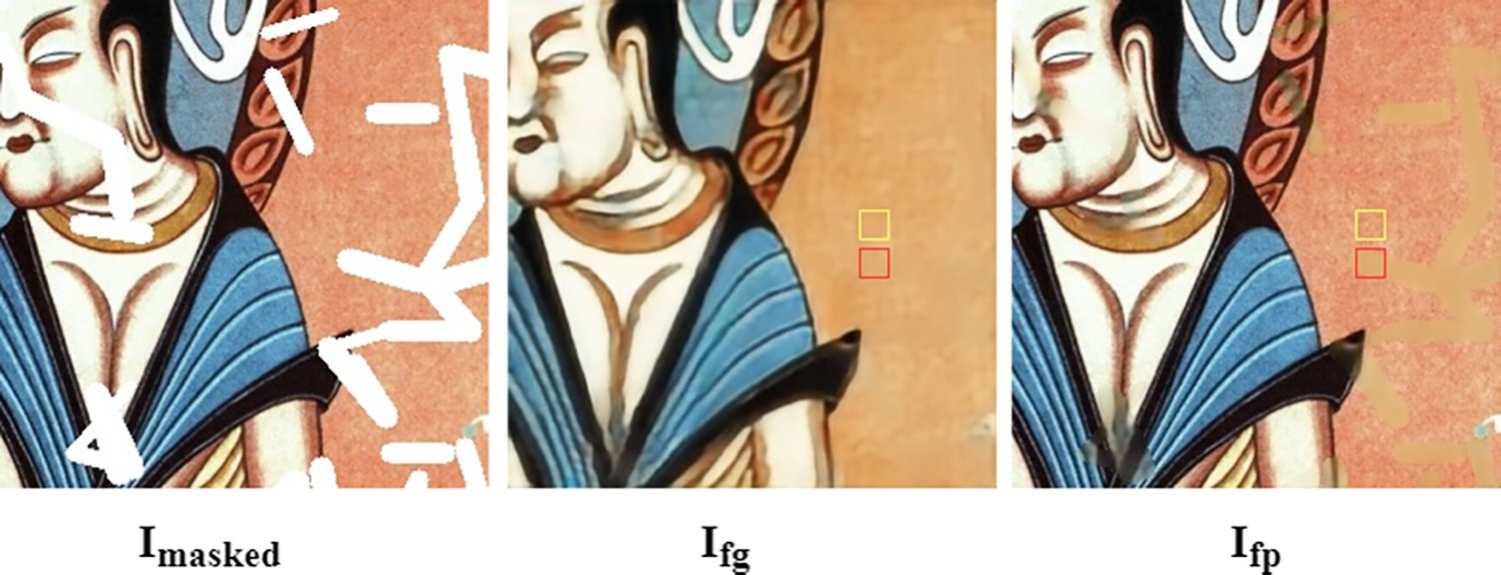
Problems in calculating the similarity with the CA method.

The SCA mechanism proposed in this paper includes three parts: attention calculation, attention propagation and feature reconstruction. Regarding the attention calculation, the existing CA and CSA methods obtain the attention score by calculating the similarity between the coarse repair result of the missing area and the cosine of the true texture of the known area, which imposes high constraints on the one-stage repair results. However, when the coarse inpainting result differs from the real texture, the calculated similarity does not accurately represent the correlation between the missing and known regions, which may lead to texture deviations in the final repaired result. Fig 2 shows I_fp_, with the red frame representing the generated result and the yellow frame representing the known true textual information. The attention score obtained by calculations based on I_fp_ do not accurately reflect the real relationship between the two regions. In contrast, the red and yellow regions in I_fg_ are generated by G1; therefore, the attention score calculated based on I_fg_ accurately represents the relationship between the missing and known regions.

**Fig 3:**
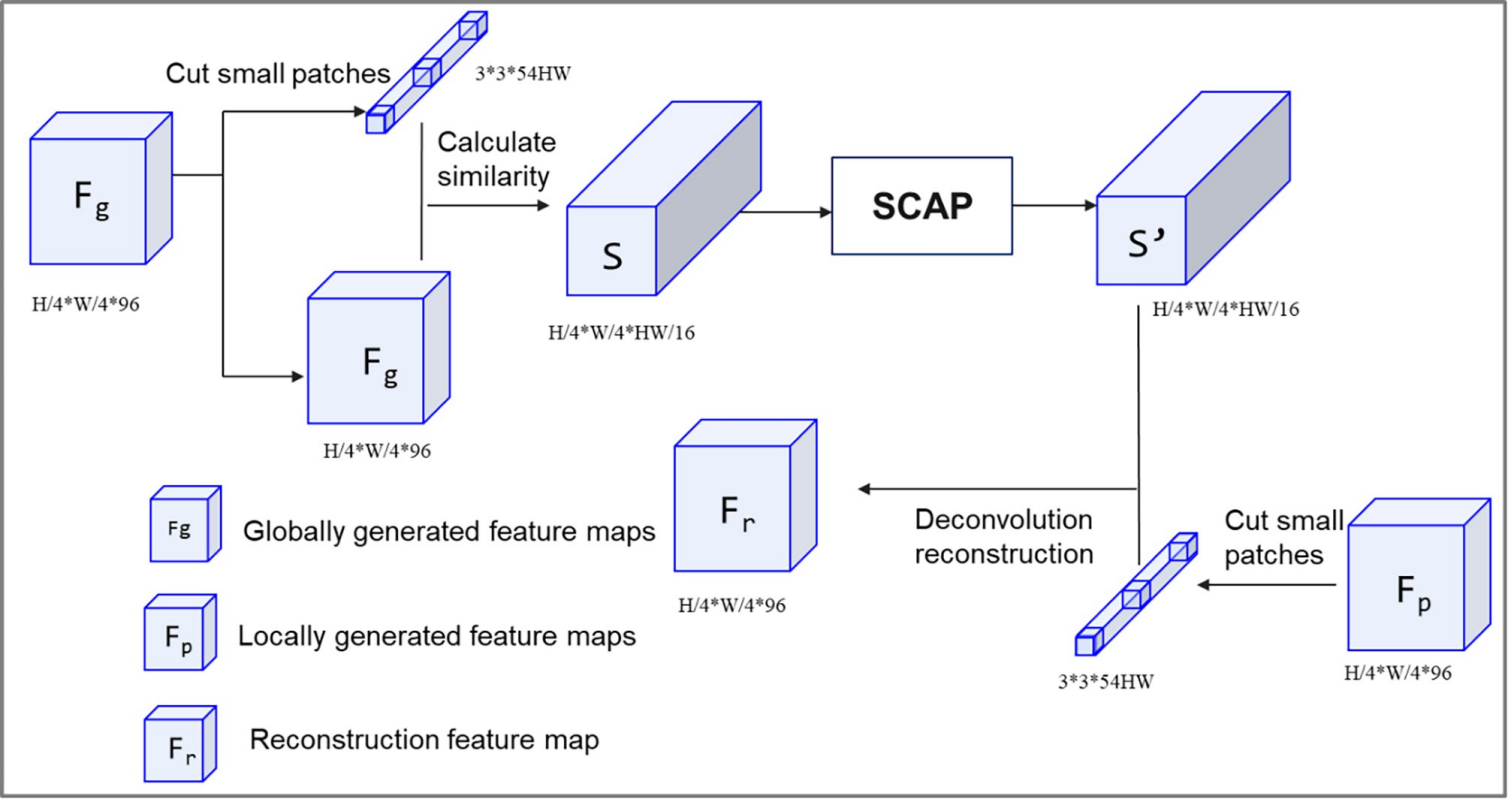
SCA structure.

To address these problems, the SCA module is proposed, as shown in Fig 3. F_g_, with a size of 32×32×192, is the feature map of I_fg_ after processing by two convolutions and nearest neighbor interpolation. F_p_, which also has a size of 32×32×192, is the feature map of I_fp_ generated after processing by two convolutions and nearest neighbor interpolation. The torch.nn.Unfold() function is utilized to extract F_g_ to obtain information block P, with a size of 3×3×192×1024, and the cosine similarity between the missing region and all known regions is calculated as follows:

$$S_{i,j}^{l}=\langle \frac{C_{i,j}}{\left\|C_{i,j}\right\|},\frac{p_{i,j}^{l}}{\left\|p_{i,j}^{l}\right\|}\rangle$$ (10)

where $p_{i,j}^{l}$ is the first feature block with a size of 3×3×192 extracted from feature map F_g_, with (i, j) as the center point, C_i,j_ is the feature block extracted from F_g_ through a sliding window during the convolution, with (i, j) as the center point, and S is the attention map with a size of 32×32×1024, which contains the similarity information of all the feature blocks. The superscript *l* denotes the *l*th layer. After the attention score is calculated, it is propagated and updated by the SCAP module, and a channel softmax normalization operation is performed to obtain the final attention score S’. The channel normalization operation is formulated is as follows:

$$s_{i,j}^{l}=\frac{\exp(s_{i,j}^{l})}{\sum_{i=1,j=1}^{N}\exp(s_{i,j}^{l})}$$ (11)

In the feature reconstruction part, F_p_ is divided into blocks, and the area to be repaired is set to 0 using the mask I_m’_ to obtain a feature block that contains only the known areas, which has a size of 3×3×1024. This feature block is used as a convolutional kernel to deconvolute S’ to obtain the reconstructed feature map F_r_, with a size of 32×32×192.

#### The SCAP module

**Fig 4:**
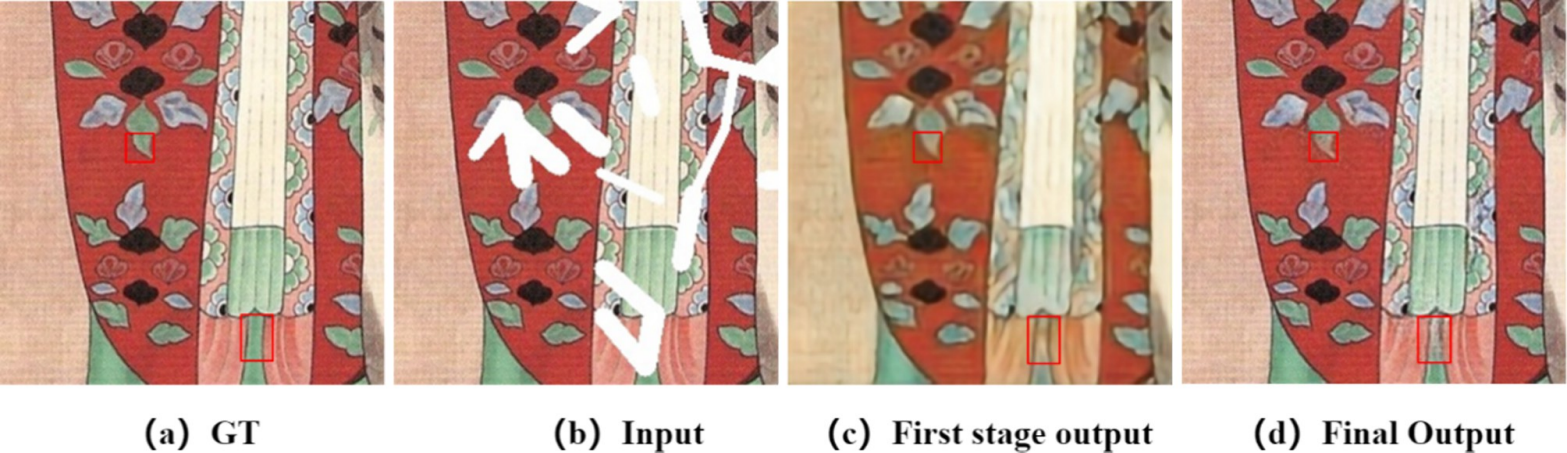
Example of image repair errors.

Unreasonable repair results during the coarse repair phase could affect the block similarity score, which could impact the final repair results. Fig 4C shows the coarse repair result. The coarse repair error in the red box region leads to increased focus on the information in the left and right regions, rather than that in the lower region, in the fine repair stage, resulting in an unreasonable final output. To address this problem, the proposed method includes an edge-constrained attention propagation module after the attention score is calculated. It is assumed that objects in the same closed area exhibit similar textures, and the attention score is updated according to edge information constraints to improve the rationality and continuity of the generated texture and improve the final repair effect. Using right propagation as an example, the similarity score can be obtained as follows:

$${\hat{S}}_{x,y,x^{\prime },y^{\prime }}=\frac{\sum_{i\in \{0,\ldots k\}}(\prod_{i=0}^{k}m_{x+i,y})•S_{x+i,y,x^{\prime },y^{\prime }}^{*}}{n}$$ (12)

where S_x,y,x’,y’_ denotes the similarity score between P_x,y_ and P_x’,y’_, and m denotes the edge information graph, which contains only two values, 0 and 1. If the value is 0, the position contains edge information. When an edge is encountered, the value on the right side is no longer considered by the model.

**Fig 5:**
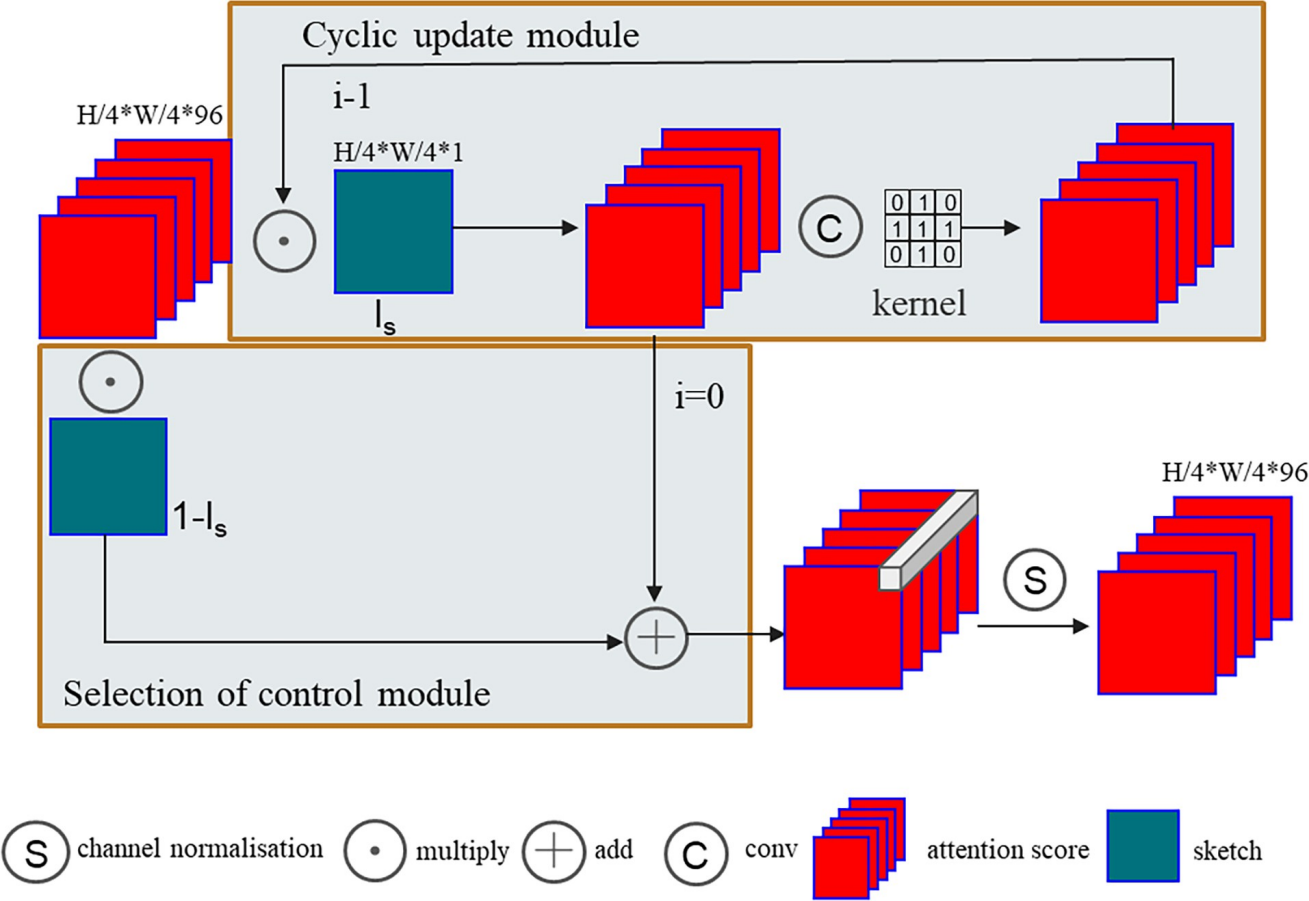
SCAP structure.

A framework diagram of the SCAP module is shown in Fig 5. The input to the model is the original attention score, and the output is the updated attention score. In the loop update module, the attention score is updated by using the surrounding information within the same closed interval. I_s_ denotes a matrix with the same size as the attention score obtained by sampling via bilinear interpolation of the structural information graph, where edge structure positions are denoted by 0, and nonedge information is denoted by 1. The attention score is stratified through element-by-element point multiplication with I_s_, and the new attention score is convolved with the convolution kernel in the graph. This convolution can be combined with the attention score contribution of the information around the defect area to update the attention score. The I_s_ function reduces the influence of edge information and surrounding nonsimilar objects during the convolution process, and the parameter *i* controls the scope of the surrounding areas in the attention update process. The selection control module combines the initial attention score of the edge position with the updated attention score of the nonedge position to update the score for only the nonedge position. Finally, the softmax function is used to obtain the final attention score. In this paper, multiple ablation experiments with different numbers of cycles are performed based on a mural dataset. The experimental results are shown in Table 2.

**Table 2:** Results for different values of i.

i	1	2	3	4
PSNR	29.9071	30.0183	30.1716	29.0163

### Algorithm steps and processes

The specific training process of the two-stage mural restoration algorithm based on the edge-constrained attention mechanism is shown in Algorithm 1.

### Algorithm 1. Training process description

**Require:** Damaged mural image, random mask, structural information map

**Ensure:** The optimal restoration model

1: Concatenate the channels of the input images.

2: **if** stage = = 1 **then**

3: Set the number of epochs to 200, with 700 iterations per epoch, using loss *L*_*cora*_

4: train G1 and D

5: **else**

6: Set the number of epochs to 200, with 700 iterations per epoch, using loss *L*_*refi*_

7: train G2 and D

8: **end if**

9: Fine-tune the model using the validation set.

10: **return** Trained model

The edge-constrained attention propagation algorithm is shown in Algorithm 2, as follows.

### Algorithm 2. SCAP algorithm for attention propagation

**1** function SCAP (x, y, i);

**Input:** x: original attention scores map, y: sketch map, i: number of loops

**Output:** a: refined attention scores map

**2** a = x

**3 if** i>0 **then**

**4** x = padding(x)

**5** x = conv(x, e)

**6** x = x*y

**7** i = i-1

8 else

**9** a = a+x*(1-y)

**10** return (a)

11 end

## Results and discussion

### Experimental environment and design

The experimental environment was based on the Windows 10 operating system, and PyTorch version 3.9.0 was used for network training. The computer was configured with an Intel (R) Core (TM) i7-11800H CPU, with 16 GB of memory. The graphics card was an NVIDIA GeForce·GTX 3060 card with 8 GB of memory. The CUDA version was 11.0, and the Adam optimizer was used for training. The training process was divided into three stages. First, the one-stage generator and discriminator were trained. The batch size was set to 4, the number of epochs was set to 100, each round included 700 iterations, and the learning rate was set to 0.001. Then, the one-stage generator was updated, and the two-stage generator and discriminator were trained. In this process, the batch size was set to 4, the number of epochs was set to 100, each round included 700 iterations, and the learning rate was set to 0.0005. Finally, the two generators and the second-stage discriminator were combined for training. The batch size was set to 4, the number of epochs was set to 20, each round included 700 iterations, and the learning rate was set to 0.0002.

There are few complete or well-preserved murals that can be used to train deep neural models because of the large damaged areas in real murals. In our study, in addition to real murals, we collected mural replicas provided by artists. The images of the real murals were obtained with a digital camera, and the mural replicas were obtained through the Complete Collection of Dunhuang Murals in China and Tomb Murals of the Silk Road in China. Our dataset included 1519 physical murals and 1295 replicas, for a total of 2814 images. To ensure that the model fully learned the relationship between the structural information and the images, we randomly cropped the images in the dataset and generated masks for data enhancement during training. Most of the structural information graphs for training and testing were assessed with the Canny edge algorithm, where the low threshold was set to 80, the high threshold was set to 240, a 3×3 Gaussian filter was used as the Sobel operator, and the binary threshold was set to 0.3. In addition, some of the sketches used in this study were manually drawn.

### Experimental analysis and comparison

To better verify the advanced nature and application value of this model, five analyses are presented in this paper: a quantitative comparative analysis, a qualitative comparative analysis, a comparative analysis of the structure repair results for different sketches, a module effectiveness analysis, and a comparative analysis of real repairs for damaged murals. To better display the structural information in the sketches, the sketches shown in the following experimental results were obtained by inverting the pixel values of the edge detection image.

### Quantitative comparative analysis

To verify the validity and versatility of the proposed model, comparison experiments are performed with datasets with four mask ratios of 10–20%, 20–30%, 30–40% and 40–50% [30]. In this paper, the peak signal-to-noise ratio (PSNR), structural similarity index measure (SSIM), and image similarity (mean square error, MSE) are used to evaluate the pixel-level difference, overall similarity, and minimization error, respectively, between the repaired and original images. The higher the PSNR and SSIM are, the better the repair effect, and the lower the MSE is, the better the repair effect [31,32]. Table 3 shows the quantitative comparison results. The proposed method ranked among the top two approaches in terms of the PSNR, SSIM and MSE metrics in the experiments with different mask ratios. These results show that the proposed method provides a better repair effect in terms of contrast, structure and quality. In addition, the experimental data reveal that the higher the mask ratio is, the larger the SSIM difference between the proposed model and the other models. Moreover, our model obtains the best PSNR. This demonstrates that compared with the other methods, the proposed method provides better structural repair effects in the restoration of murals with large damaged areas while ensuring high image quality.

**Table 3:** Comparison of the results for different mask ratios.

Index	Mask ratio			
	10–20%	20–30%	30–40%	40–50%
**PSNR**				
DeepFillV2 [17]	35.03	28.97	24.29	22.87
RFR [22]	34.34	28.40	24.98	22.95
SC-FEGAN [33]	33.45	27.58	23.34	21.59
Lama [34]	34.20	28.44	25.27	23.16
SketchRefiner [35]	35.11	30.02	25.67	24.53
Our approach	36.26	30.11	26.00	24.22
**SSIM**				
DeepFillV2 [17]	0.9834	0.9692	0.9453	0.9292
RFR [22]	0.9773	0.9628	0.9435	0.9179
SC-FEGAN [33]	0.9695	0.9672	0.9378	0.9277
Lama [34]	0.9880	0.9722	0.9466	0.9297
SketchRefiner [35]	0.9870	0.9762	0.9489	0.9388
Our approach	0.9872	0.9753	0.9510	0.9395
**MSE**				
DeepFillV2 [17]	0.0051	0.0120	0.0218	0.0314
RFR [22]	0.0056	0.0118	0.0211	0.0298
SC-FEGAN [33]	0.0045	0.0122	0.0250	0.0352
Lama [34]	0.0056	0.0118	0.0215	0.0298
SketchRefiner [35]	0.0053	0.0102	0.0210	0.0290
Our approach	0.0045	0.0106	0.0201	0.0278

### Qualitative comparative analysis

**Fig 6:**
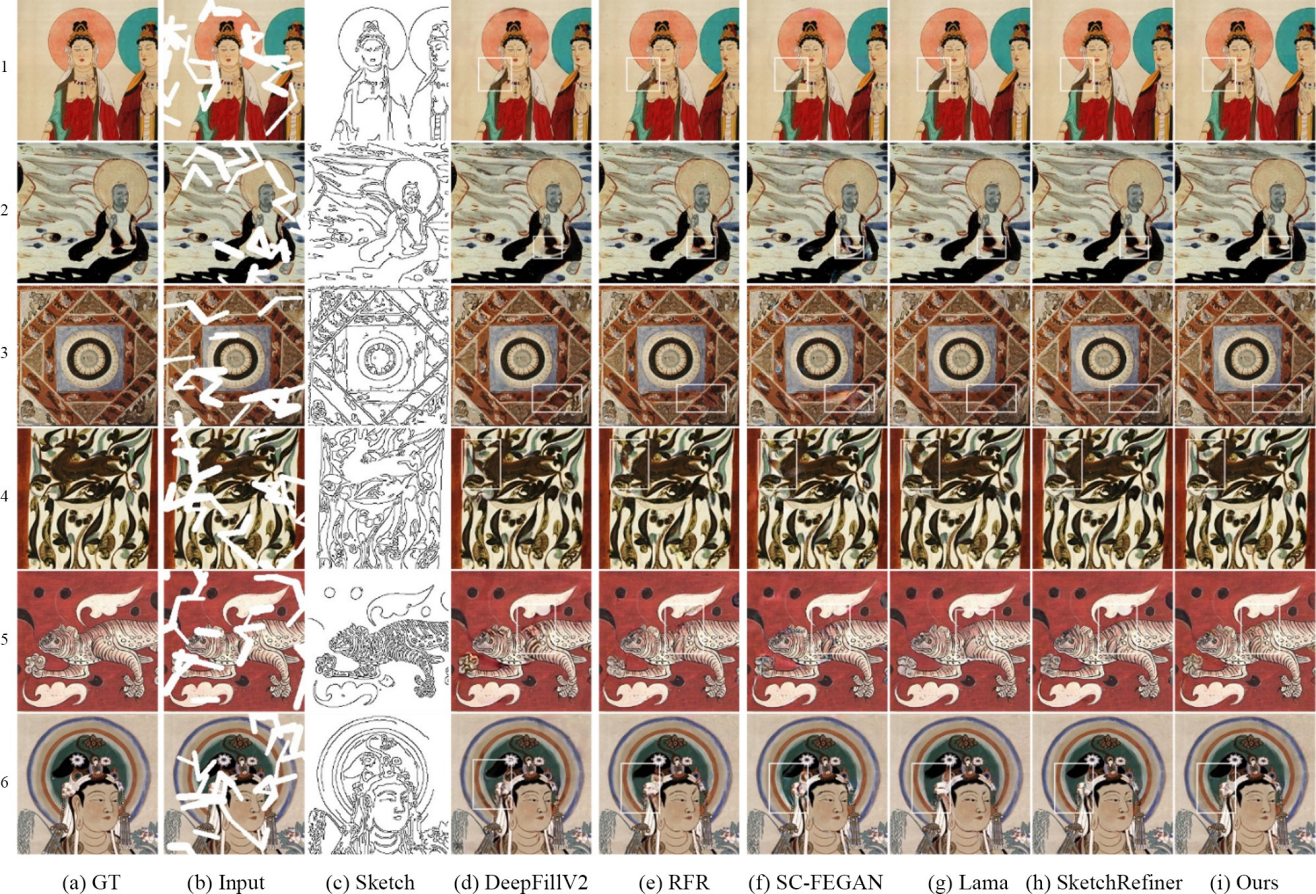
Comparison of the mural restoration results obtained by the different models.

To subjectively evaluate the repair effect of the proposed model, qualitative experiments are conducted on the basis of the sketch obtained by the Canny edge detection algorithm. The comparison results are shown in Fig 6. The RFR model obtains realistic texture details via circular feature inference; however, this method does not consider the guiding effect of the sketches, which causes the results to deviate from the original structure. Although there are no obvious visual errors in the silk image in Fig 6–1(E), the deer leg image in Fig 6–4(E), the auspicious cloud image in Fig 6–5(E), and the screw bun image in Fig 6–6(E), the generated structures greatly differ from the real structure. Compared with the RFR model, the Lama method generates both realistic textures and visually reasonable structural information for most images. Nevertheless, there are some differences between the structures generated by the Lama method and those of the real images (e.g., the deer leg shown in Fig 6–4(G)). The DeepFillV2 model uses sketch guidance to generate the desired information. However, the DeepFillV2 model uses a contextual attention mechanism in the second repair stage. Thus, when this method is used to repair mural images with rich colors, the repaired result significantly differs from the real image, which has a smooth texture. For example, Fig 6–5(D) shows a tiger image with a red background, and Fig 6–6(D) shows a Buddha head light image. The texture of the repair result significantly deviates from the surrounding real texture. In addition, the sketch-guided SC-FEGAN method does not completely repair some structures and does not fully utilize the surrounding texture information, leading to inaccurate colors in the repair result. As shown in Fig 6–2(F) and 6–3(F), there are obvious visual artifacts in the white frame area. The SketchRefiner method generates not only correct structural information for most images but also detailed textures. However, some of the repair results show unnatural structures and different textures than the actual results. For instance, the white box area in Fig 6–2(H) is gray, while the original image is black, and the deer antler lines in Fig 6–4(H) are slightly blurry. The proposed method generates accurate and clear structures, and the generated texture is essentially consistent with the real texture. These results verify that the proposed method can improve the inpainting effect for mural images.

### Comparative analysis of the repair results with different sketch structures

**Fig 7:**
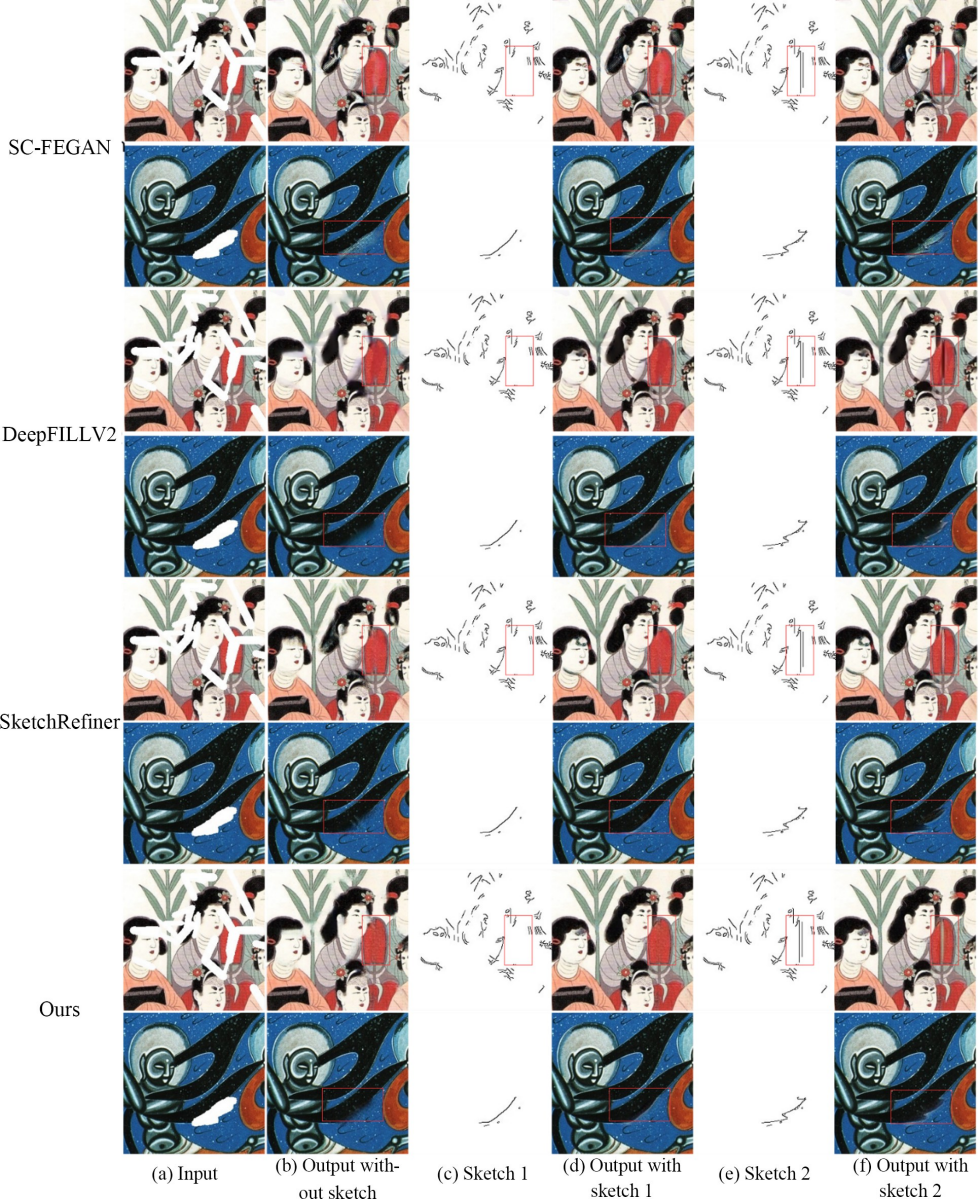
Results for the different sketches.

To verify the importance of sketch information in the repair process and evaluate the repair effect of the model guided by different sketch structures, a comparative experiment is performed in this paper according to the presence or absence of sketches and the use of different structural sketches. Fig 7 shows the different parts of sketches 1 and 2, which were manually drawn. Fig 7B shows that without the guidance of the sketch structure, the four repair methods can obtain suitable results in unstructured regions with the same surrounding texture, but the results are blurred at the boundary between regions with different textures. Thus, sketches are crucial for the structural restoration of mural images. Fig 7D and 7F reveal that the DeepFillV2 and SC-FEGAN models both generate simple structures based on the sketch information. However, the texture quality generated by the DeepFillV2 model considerably differs from the real texture, while the results of the SC-FEGAN model contain visual artifacts. For complex sketch structures, the SC-FEGAN model produces structural errors in the repaired regions, while the DeepFillV2 model generates incomplete structural information. Although SketchRefiner can generate complete structures according to the sketch information, the method proposed in this study achieves a better effect: the generated structure information is clearer and more accurate, with a better texture repair effect.

### Module effectiveness analysis

We also conducted three decomposition experiments based on the mural dataset to verify the effectiveness of our proposed modules. The image restoration results of all the experiments are randomly generated masks.

**Fig 8:**
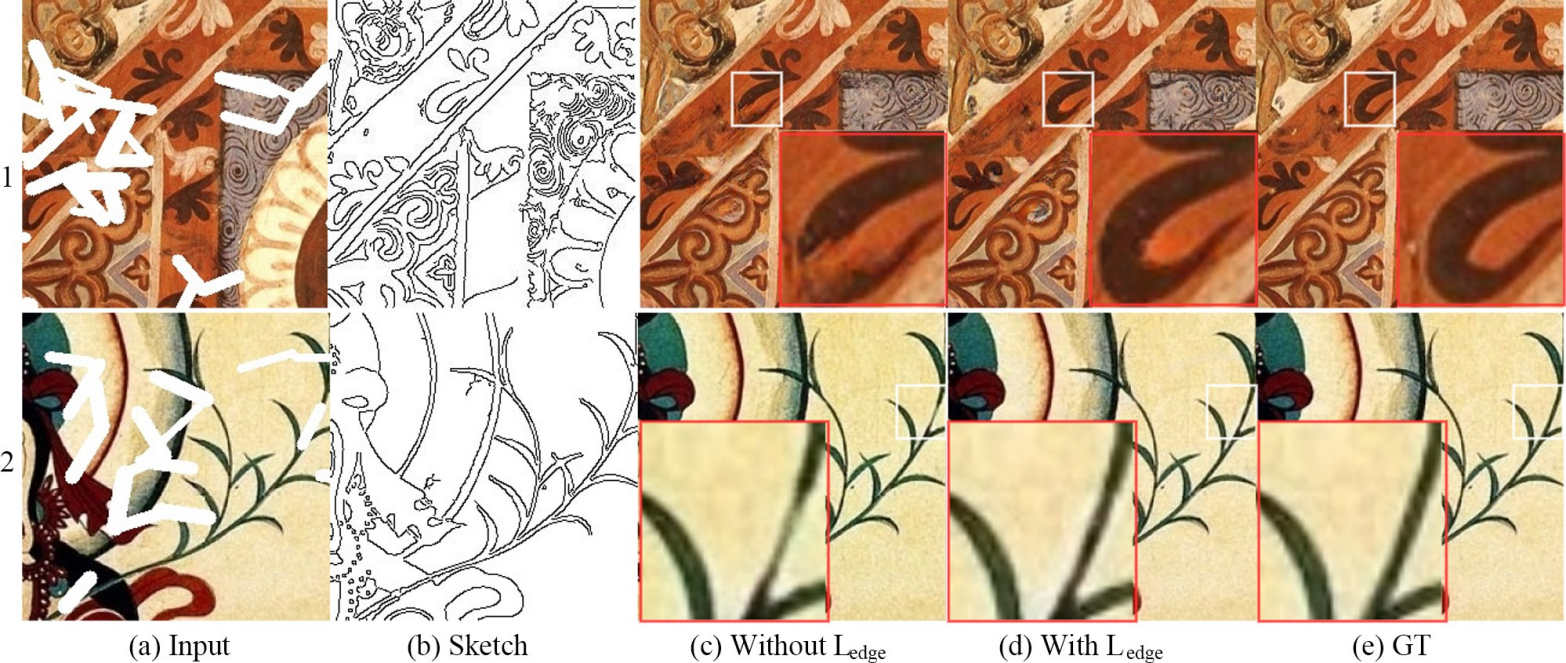
Results with and without L_edge_.

**Fig 9:**
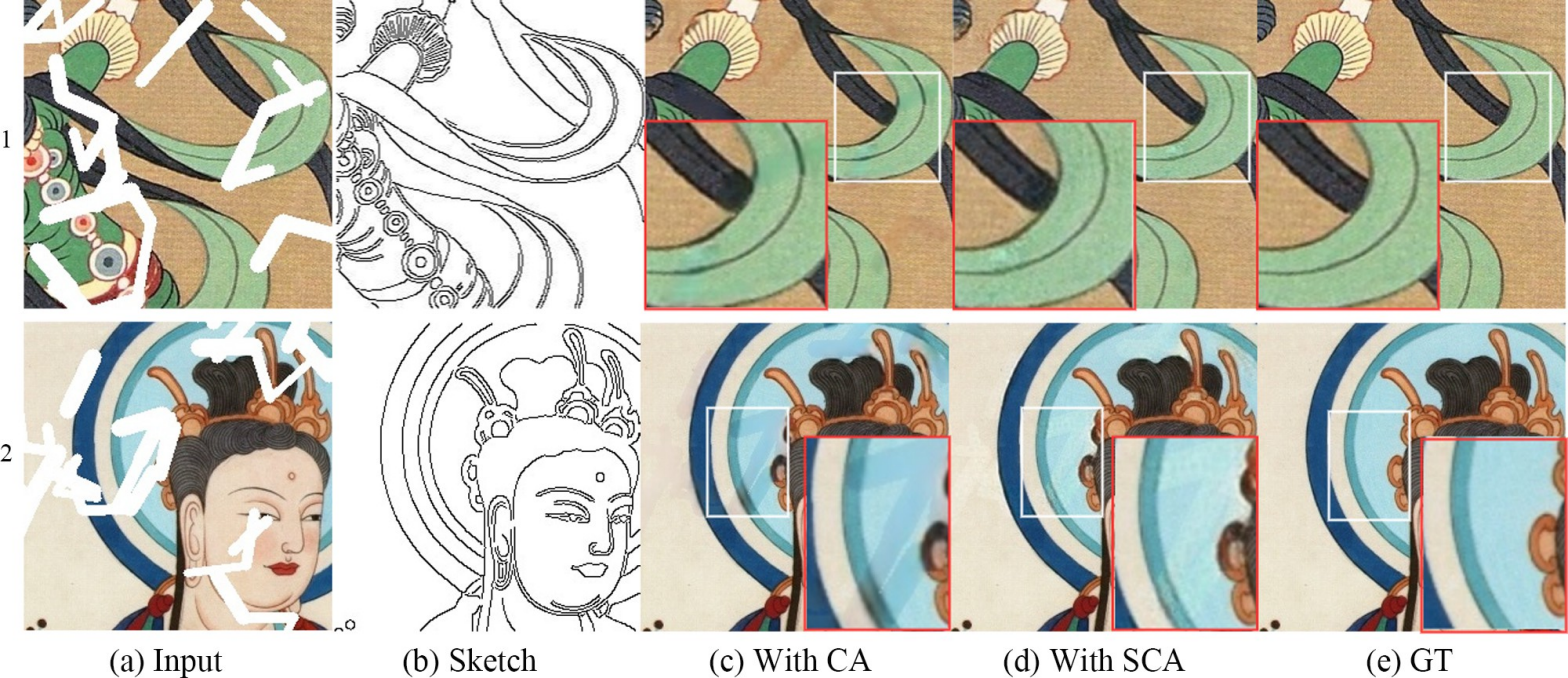
Results with the CA and SCA modules.

**Fig 10:**
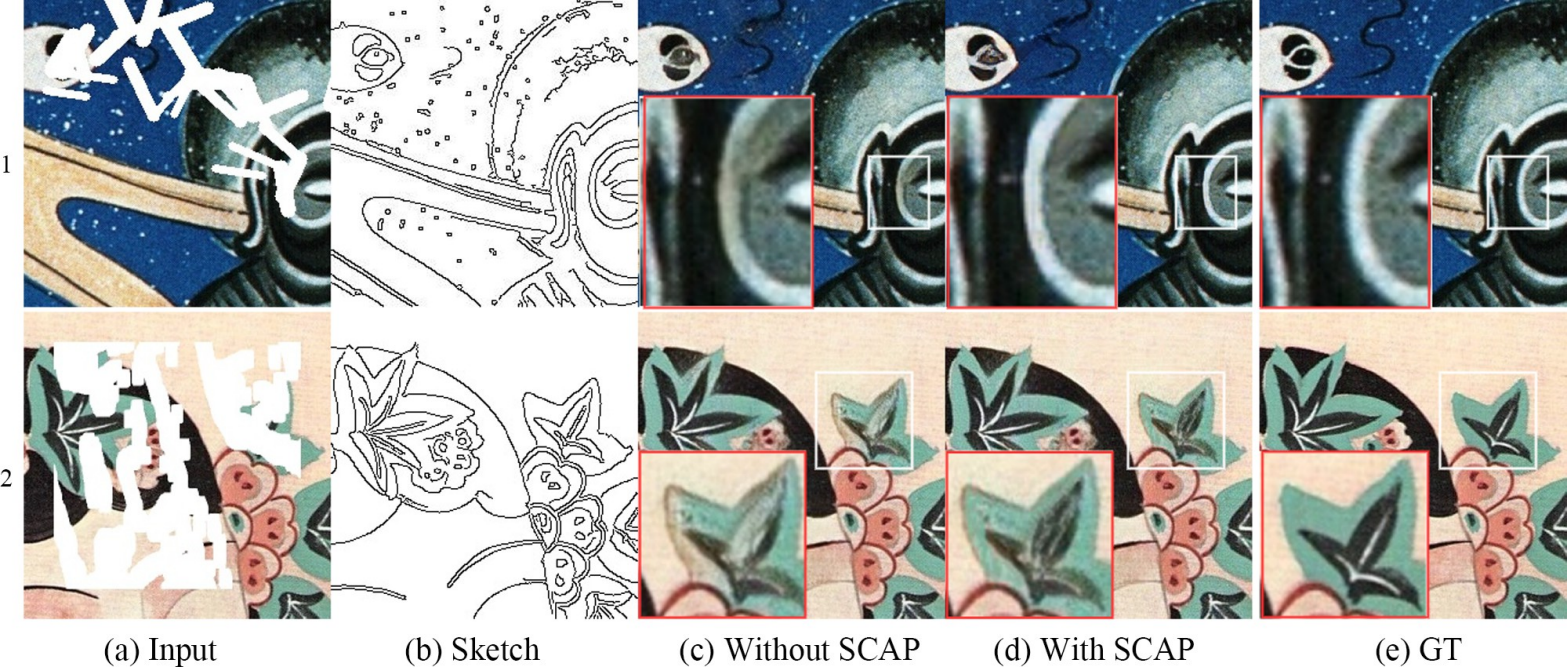
Results with and without the SCAP module.

1. Effectiveness of the L_edge_ function
  Fig 8A shows the input image to be repaired, Fig 8B shows the structure sketch extracted from the original image, Fig 8C shows the image inpainting effect without the L_edge_ loss function, Fig 8D shows the image inpainting effect with the L_edge_ loss function, and Fig 8E shows the original image. The comparative analysis demonstrates that the L_edge_ function proposed in this paper improves the structural reconstruction ability of the proposed model.
2. Effectiveness of the SCA module
  Fig 9A shows the input image to be repaired, Fig 9B shows the structure sketch extracted from the original image, Fig 9C shows the image inpainting result of the mainstream CA algorithm, Fig 9D shows the image inpainting result of the SCA-based image inpainting module proposed in this paper, and Fig 9E shows the original image. The texture of the repair result obtained using the CA method significantly deviates from the real texture, whereas this texture deviation is hardly visible in the repair result obtained using the SCA method. This shows that the SCA method achieves better texture reconstruction ability based on the mural dataset than previous approaches.
3. Effectiveness of the SCAP module
  Fig 10A shows the input image to be repaired, Fig 10B shows the structure sketch extracted from the original image, Fig 10C shows the image restoration effect without the SCAP module, Fig 10D shows the image restoration effect with the SCAP module, and Fig 10E shows the original image. There are color errors in the repair results of the network without the SCAP module, while the network with the SCAP module propagates and updates the attention scores between similar objects and thus generates the correct color.

## Repair analysis of real damaged murals

**Fig 11:**
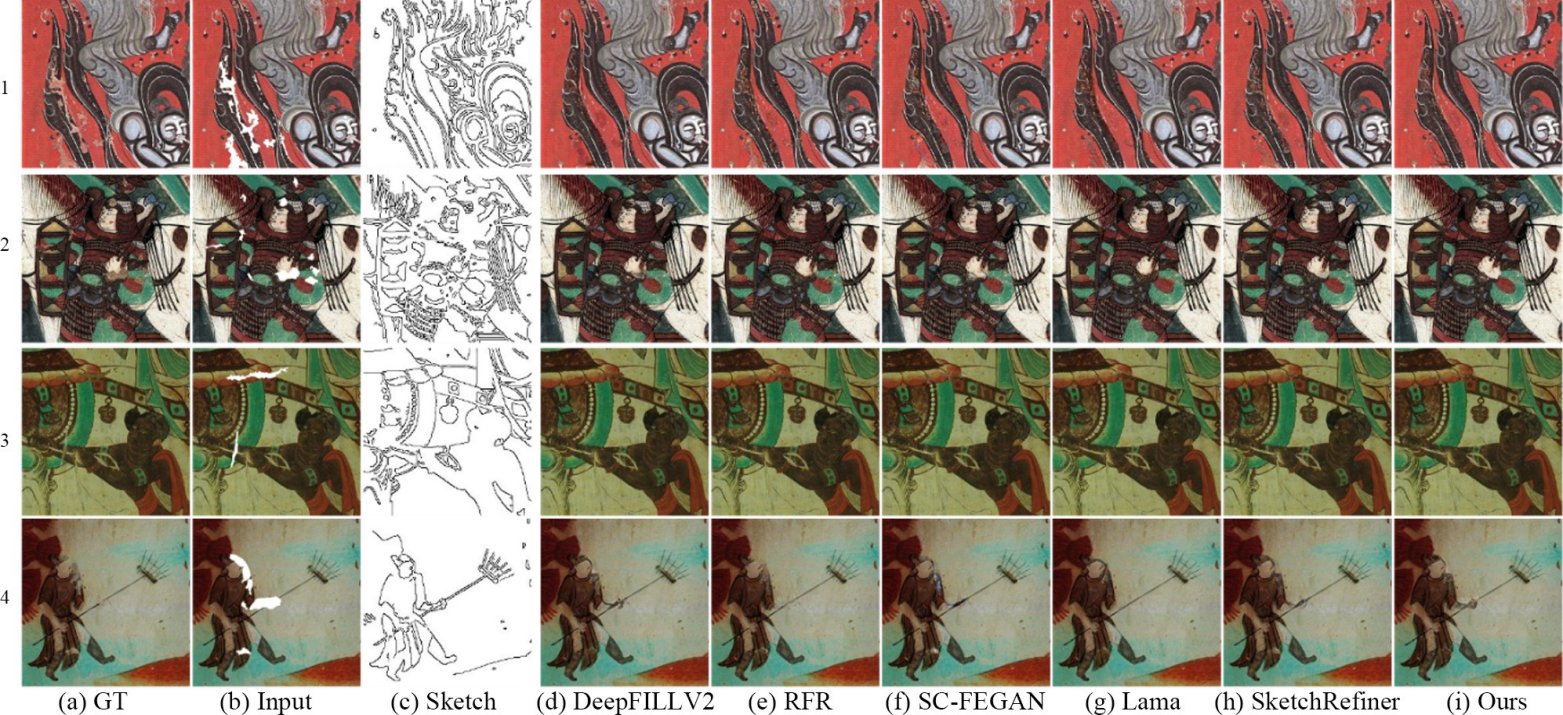
Results for real damaged murals.

To further verify the feasibility of the proposed model, four groups of real damaged mural images are analyzed via repair experiments. We marked the defect area, extracted the structural information of the nondefect area, and manually determined the structure of the defect area. The repair results are shown in Fig 11. The proposed method generates a reasonable structure according to the sketch information, and the generated texture and color are consistent with those of the original mural. The repair effect of the proposed method is better than that of the other three mainstream methods. Therefore, the proposed method has considerable application value in mural image restoration.

**Fig 12:**
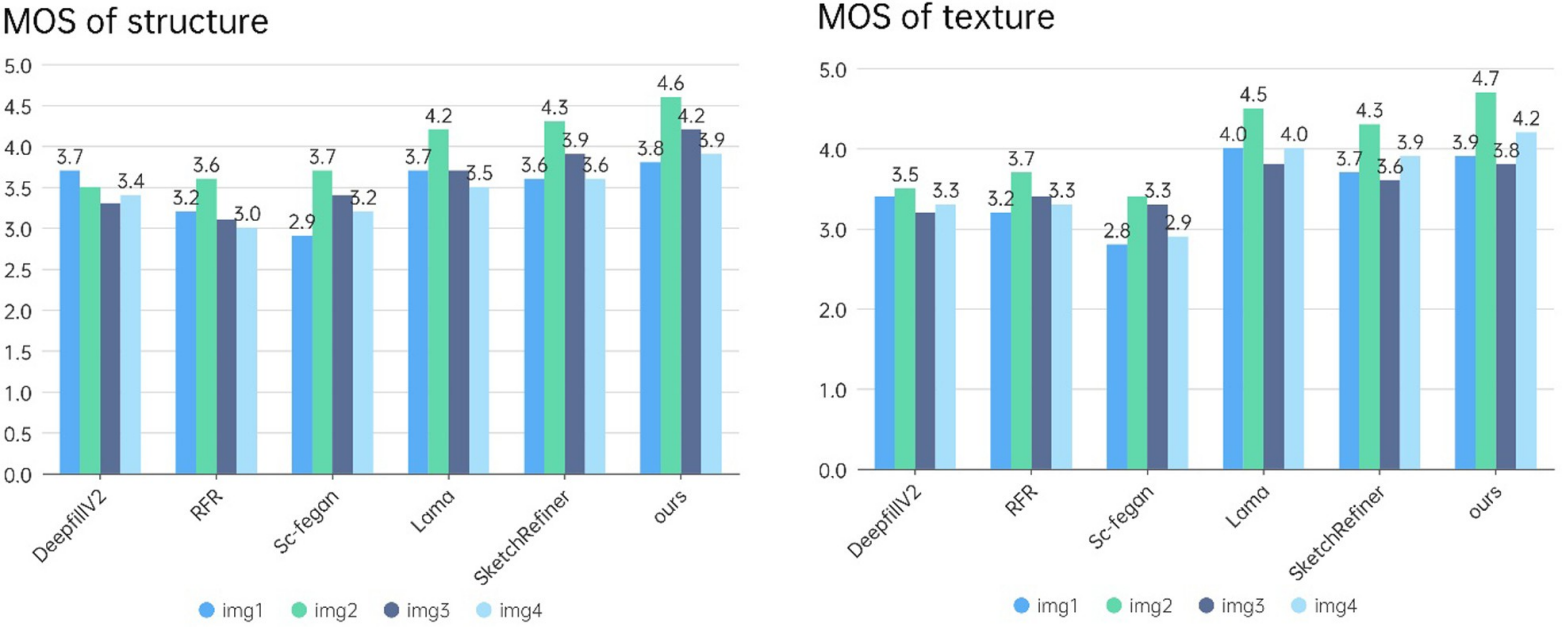
Comparison of the subjective and objective evaluation scores of the restoration results for real damaged murals. (a) shows the MOS value of the similarity between the structure and sketch, and (b) shows the MOS value of the texture restoration result.

Due to the lack of corresponding standard reference images for real damaged murals, a nonreference image quality assessment method is employed to evaluate the repair results. The mean opinion score (MOS) without a reference evaluation index is used. The MOS is a quality score representing the observer’s judgment of the structural and textural repair effects according to set evaluation criteria [29,30]. The higher the MOS value is, the better the repair effect. The corresponding relationships between the MOS value and the restoration effect are summarized in Table 4. The results of the real damaged mural restoration experiment shown in Fig 11 are evaluated according to the MOS, and the results are shown in Fig 12. The MOS evaluation results for the structure and texture of the images generated by the proposed method are better than those of the images generated by the comparison methods, indicating that the proposed method can generate reasonable structural information and textures that are more consistent with the real textures. The findings suggest that the proposed method outperforms the comparison methods in terms of both subjective and objective evaluation indices, thus verifying the effectiveness and suitability of the proposed method for the restoration of real murals.

**Table 4:** Subjective evaluation and quantitative results for the restoration of real damaged murals.

Quality	Similarity between the structure and sketch	Texture restoration result	MOS
Excellent	Identical	Nearly impossible to detect differences	5
Good	Highly similar	Subtle traces of imperfections	4
Fair	Similar	Noticeable flaws or traces	3
Poor	Moderate similarity	Most defects still clearly visible	2
Very poor	Different	Remain highly noticeable	1

## Conclusion

In this study, a two-stage mural image restoration model based on an edge-constrained attention mechanism is designed. In this model, gated convolutions combined with a local edge loss function are introduced in the coarse repair stage to improve the guidance ability of the sketches in the feature extraction process considering network constraints and the consistency between the structural information of the user-guided sketch and that of the generated image. In the fine repair stage, an edge-constrained attention propagation module is introduced, which calculates attention based on both known and missing regions. This treatment enables the obtained attention score to better reflect the similarity degree between the missing and the know regions and thus enhances the local consistency between the generated and the know textures. The integrated edge-constrained attention propagation algorithm, following the attention mechanism, utilizes the structural information of the sketch to propagate and update the attention score and enhances the semantic relevance between the generated results and the known areas. This study leads to the following main findings:

1. Across various mask ratios, our proposed method consistently outperforms most compared methods in terms of PSNR and SSIM metrics. Specifically, at mask ratios of 10–20%, 20–30%, 30–40%, and 40–50%, the PSNR values achieved by our method are 36.26, 30.11, 26.00, and 24.22 dB, respectively. These values surpass those obtained by DeepFillV2, RFR, SC-FEGAN, and Lama. Additionally, our method demonstrates superior performance in the SSIM metric across all mask ratios, achieving 0.9872, 0.9753, 0.9510, and 0.9395, respectively.
2. When guided by user sketches, our proposed method effectively produces visually complete inpainting results that maintain structural consistency with the sketches. This finding demonstrates the method’s ability to leverage user-provided prior knowledge, ensuring the generated inpainting images meet user expectations.
3. When applied to real-world damaged mural images, our proposed method consistently outperforms competing methods based on subjective evaluation scores. This finding demonstrates the method’s effectiveness and robustness in addressing real-world damage, delivering visually satisfactory inpainting results.

The network proposed in this paper can generate results that satisfy users expectations according to provided sketches and the repaired and the known regions exhibit a high level of texture consistency, demonstrating a novel and practical mural repair method. However, this network also has limitations. The proposed attention propagation module uses only the information around the same object to propagate updates and does not consider the information of similar objects, such as repeated patterns and decorations in clothing. Particularly, when patterns and decorations are missing, the final repair result may exhibit texture errors. To further improve the effectiveness of the proposed model, multiscale information should be considered in the attention module to better capture the correlations between similar objects. Furthermore, the attention propagation algorithm can be further optimized to propagate information based on distant but similar objects. Additionally, mural datasets can be enriched to enable the network to learn more semantic information in the coarse repair stage.

## References

[1] Ha YP, McDonald N, Hersh S, Fenniri SR, Hillier A, Cannuscio CC. Using informational murals and handwashing stations to increase access to sanitation among people experiencing homelessness during the COVID-19 pandemic. Am J Public Health 2021;111(1):50–53. doi: 10.2105/AJPH.2020.305961 33211587 PMC7750595

[2] Mural celebrates staff and diversity in the NHS. Nursing Management 2020;27(6).

[3] Mendelson-Shwartz E, Mualam N. Taming murals in the city: A foray into mural policies, practices, and regulation. Int J Cult Policy 2021;27(1):65–86.

[4] Shao H, Wang YX. Generative image inpainting with salient prior and relative total variation. J Vis Commun Image Represent 2021;79:103231.

[5] Brkic AL, Mitrovic D, Novak A. On the image inpainting problem from the viewpoint of a nonlocal Cahn-Hilliard type equation. J Adv Res 2020;25:67–76. doi: 10.1016/j.jare.2020.04.015 32922975 PMC7474195

[6] Yang XH, Guo BL, Xiao ZL, Liang W. Improved structure tensor for fine-grained texture inpainting. Signal Processing: Image Communication 2019;73:84–95.

[7] Fan Y. Damaged region filling by improved criminisi image inpainting algorithm for thangka. Cluster Computing 2019;22(6):13683–13691.

[8] Pathak D, Krahenbuhl P, Donahue J, Darrell T, Efros AA. Context encoders: Feature learning by inpainting. Proceedings of the IEEE conference on computer vision and pattern recognition. 2016;2536–2544.

[9] Iizuka S, Simo-Serra E, Ishikawa H. Globally and locally consistent image completion. ACM Transactions on Graphics (ToG) 2017; 36(4): 1–14.

[10] Song Y, Yang C, Lin Z, Liu X, Huang Q, Li H, et al. Contextual based image inpainting: Infer, match, and translate. In: Proceedings of the 15th European Conference on Computer Vision. Munich, Germany: Springer, 2018; 3−18.

[11] Yan Z, Li X, Li M, Zuo W, Shan S. Shift-net: Image inpainting via deep feature rearrangement. Proceedings of the European conference on computer vision (ECCV). 2018;1–17.

[12] Ronneberger O, Fischer P, Brox T. U-net: Convolutional networks for biomedical image segmentation. International Conference on Medical image computing and computer-assisted intervention. Springer, Cham, Munich, Germany. 2015;234–241.

[13] Yu J, Lin Z, Yang J, Shen X, Lu X, Huang TS. Generative image inpainting with contextual attention. Proceedings of the IEEE Conference on Computer Vision and Pattern Recognition. 2018;5505–5514.

[14] Liu H, Jiang B, Xiao Y, Yang C. Coherent semantic attention for image inpainting. //Proceedings of the IEEE/CVF International Conference on Computer Vision. 2019;4170–4179.

[15] Nazeri K, Ng E, Joseph T, Qureshi FZ, Ebrahimi M. Edgeconnect: Generative image inpainting with adversarial edge learning. ICCV Workshops. 2019;arXiv:1901.00212.

[16] Portenier T, Hu Q, Szabó A, Bigdeli SA, Favaro P, Zwicker M. Faceshop: Deep sketch-based face image editing. 2018;arXiv:1804.08972.

[17] Yu J, Lin Z, Yang J, Shen X, Lu X, Huang TS. Free-form image inpainting with gated convolution. The IEEE/CVF International Conference on Computer Vision. 2019;4471–.

[18] Ren JL. Research on Tangka image restoration based on multi-scale attention mechanism and edge constraints (dissertation). Ningxia University, 2018.

[19] Liu W, Shi Y, Li J, Wang J, Du S. Multi-stage progressive reasoning for Dunhuang murals inpainting. In: 2023 IEEE 4th International Conference on Pattern Recognition and Machine Learning (PRML). IEEE, 2023: 211–217.

[20] Chen Y, Xia R, Yang K, Zou K. DNNAM: Image Inpainting Algorithm via Deep Neural Networks and Attention Mechanism. Applied Soft Computing 2024: 111392.

[21] Cao C, Dong Q, Fu Y. Learning prior feature and attention enhanced image inpainting. In European Conference on Computer Vision, Cham, Springer Nature Switzerland; 2022, pp. 306–322.

[22] Li J, Wang N, Zhang L, Du B, Tao D. Recurrent feature reasoning for image inpainting. Proceedings of the IEEE/CVF Conference on Computer Vision and Pattern Recognition. 2020;7760–7768.

[23] Cao CR, Liu WR, Shi CH, Zhang HC. Generative image inpainting with attention propagation. Acta Automatica Sinica 2022; 48(05):1343–1352 (in Chinese with an English abstract).

[24] Chen WT, Xia RL, Yang K, Zou K. MFMAM: Image inpainting via multi-scale feature module with attention module. Computer Vision and Image Understanding, 2024; 238:103883.

[25] Salimans T, Goodfellow I, Zaremba W, Cheung V, Radford A, Chen X. Improved techniques for training gans. Advances in neural information processing systems. 2016;29:2234–2242.

[26] Zhu JY, Park T, Isola P, Efros AA. Unpaired image-to-image translation using cycle-consistent adversarial networks. Proceedings of the IEEE international conference on computer vision. Piscataway: IEEE. 2017;2223–2232.

[27] Sajjadi MSM, Scholkopf B, Hirsch M. Enhancenet: Single image super-resolution through automated texture synthesis. Proceedings of the IEEE International Conference on Computer Vision. Piscataway: IEEE. 2017;4491–4500.

[28] Russakovsky O, Deng J, Su H, Krause J, Satheesh S, Ma S, et al. Imagenet large scale visual recognition challenge. Int J Comput Vis 2015;115(3):211–252.

[29] Simonyan K, Zisserman A. Very deep convolutional networks for large-Scale image recognition. Computer Science, 2014;arXiv:1409.1556.

[30] Liu G, Reda FA, Shih KJ, Wang TC, Tao A, Catanzaro B. Image inpainting for irregular holes using partial convolutions. Proceedings of the European conference on computer vision (ECCV). 2018;85–100.

[31] Ye YQ. Design and implementation of non-reference thangka image quality evaluation system. Lanzhou: Northwest University for Nationalities, 2020.

[32] Yang X. Research on quality evaluation method of nonreference image based on convolutional neural network. Fuzhou: East China Institute of Technology, 2020.

[33] Jo Y, Park J. Sc-fegan: face editing generative adversarial network with user’s sketch and color. The IEEE/CVF International Conference on Computer Vision. 2019;1745–1753.

[34] Suvorov R, Logacheva E, Mashikhin A, Remizova A, Ashukha A, Silvestrov A, et al. Resolution-robust large mask inpainting with fourier convolutions. In: Proceedings of the IEEE/CVF winter conference on applications of computer vision. 2022; 2149–2159.

[35] Liu C, Xu S, Peng J, Zhang K, Liu D. Towards interactive image inpainting via sketch refinement. arXiv preprint arXiv:2306.00407, 2023.

